# Natural radionuclide of Po^210 ^in the edible seafood affected by coal-fired power plant industry in Kapar coastal area of Malaysia

**DOI:** 10.1186/1476-069X-10-43

**Published:** 2011-05-20

**Authors:** Lubna Alam, Che Abd Rahim Mohamed

**Affiliations:** 1 School of Environment and Natural Resource Sciences, Faculty of Science and Technology, Universiti Kebangsaan Malaysia, Bangi, 43600, Selangor, Malaysia; 2 Marine Ecosystem Research Centre (EKOMAR), Faculty of Science and Technology, Universiti Kebangsaan Malaysia, Bangi, 43600, Selangor, Malaysia

## Abstract

**Background:**

Po^210 ^can be accumulated in various environmental materials, including marine organisms, and contributes to the dose of natural radiation in seafood. The concentration of this radionuclide in the marine environment can be influenced by the operation of a coal burning power plant but existing studies regarding this issue are not well documented. Therefore, the aim of this study was to estimate the Po^210 ^concentration level in marine organisms from the coastal area of Kapar, Malaysia which is very near to a coal burning power plant station and to assess its impact on seafood consumers.

**Methods:**

Concentration of Po^210 ^was determined in the edible muscle of seafood and water from the coastal area of Kapar, Malaysia using radiochemical separation and the Alpha Spectrometry technique.

**Results:**

The activities of Po^210 ^in the dissolved phase of water samples ranged between 0.51 ± 0.21 and 0.71 ± 0.24 mBql^-1 ^whereas the particulate phase registered a range of 50.34 ± 11.40 to 72.07 ± 21.20 Bqkg^-1^. The ranges of Po^210 ^activities in the organism samples were 4.4 ± 0.12 to 6.4 ± 0.95 Bqkg^-1 ^dry wt in fish (*Arius maculatus*), 45.7 ± 0.86 to 54.4 ± 1.58 Bqkg^-1 ^dry wt in shrimp (*Penaeus merguiensis*) and 104.3 ± 3.44 to 293.8 ± 10.04 Bqkg^-1 ^dry wt in cockle (*Anadara granosa*). The variation of Po^210 ^in organisms is dependent on the mode of their life style, ambient water concentration and seasonal changes. The concentration factors calculated for fish and molluscs were higher than the recommended values by the IAEA. An assessment of daily intake and received dose due to the consumption of seafood was also carried out and found to be 2083.85 mBqday^-1^person^-1 ^and 249.30 μSvyr^-1 ^respectively. These values are comparatively higher than reported values in other countries. Moreover, the transformation of Po^210 ^in the human body was calculated and revealed that a considerable amount of Po^210 ^can be absorbed in the internal organs. The calculated values of life time mortality and morbidity cancer risks were 24.8 × 10^-4 ^and 34 × 10^-4 ^respectively which also exceeded the recommended limits set by the ICRP.

**Conclusions:**

The findings of this present study can be used to evaluate the safety dose uptake level of seafood as well as to monitor environmental health. However, as the calculated dose and cancer risks were found to cross the limit of safety, finding a realistic way to moderate the risk is imperative.

## Background

As the world's second most important energy source, coal plays a vital role in the electricity generation sector, contributing up to 39% in global electricity production [1]. Experts at the International Energy Agency (IEA) in Paris estimated that demand for coal will increase in the next two decades from the current level of about 6.7 billion tons per year to almost 10 billion tons in 2030 [2]. In the case of Malaysia, coal contributed up to 34.2% of electricity generation [3]. Coal contains minor amounts of the radioactive elements uranium and thorium which are not a problem for environment [4]. But a coal-fired power station produces electricity by burning coal, which emits fry ash as a by-product that contains uranium and thorium that is ten times higher than their original levels. In fact, the fly ash carries 100 times more radiation into the surrounding environment than a nuclear power plant producing the same amount of energy [4]. Fly ash uranium sometimes leaches into the soil and water surrounding a coal plant. As a result the radiation dose ingested by the people living near the coal plant can be equal to or higher than doses for people living around nuclear facilities [5]. Po^210 ^(T_1/2 _= 138.4 d) is an alpha emitter within the U-238 decay series and among the natural radionuclides occurring in the ocean, alpha emitters are considered to be the most important because of its radiation exposure.

This radionuclide is accumulated by a variety of marine organisms and is known to be a major contributor (90%) to the dose of natural radiation coming from alpha emitting radionuclides received by most marine organisms [6,7]. It is also a major contributor to critical group doses from seafood consumption, in particular from the consumption of molluscs [8]. Ingestion of Po^210 ^through seafood consumption varies from place to place and depends on the concentration of Po^210 ^in seafood organisms, as well as on the consumption rate of seafood.

Malaysia is among the countries with the highest fish consumption in the world and relies on seafood as a main source of animal protein. It is known to be the highest consumer of seafood in the Southeast Asian region, both in terms of per capita intake and percentage of protein. Therefore, a study regarding the Po^210 ^concentration in edible seafood is very important for this country in order to assess whether there is an existing health hazard. The objectives of this study was to examine and compare the accumulation pattern of Po^210 ^in the marine environment and three groups of seafood (fish, crustaceans and molluscs) originating from the Kapar power plant area as well as to estimate its impact on seafood consumers.

## Methods

### Study area

**Figure 1:**
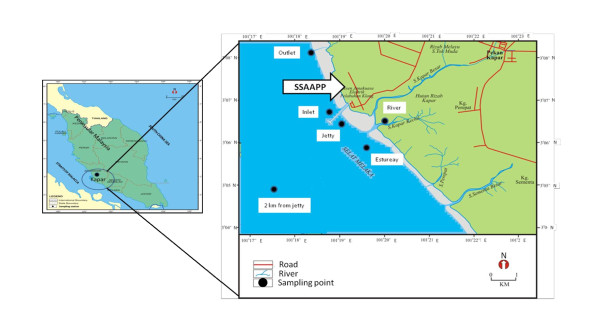
**Sampling locations at the Kapar Coastal Area**.

The Sultan Salahuddin Abdul Aziz power station, which is located on the western coast of west Malaysia, along the Malacca Straits, is recognised as the largest power station in the country with a generating capacity of 2420 MW, contributing to about 23% of the country's energy demand (Figure 1). This power station is the first to have a triple fuel firing capability (gas, oil and coal) in Malaysia. The power station lies between the mouth of the Kapar Besar and Serdang Kecil rivers and is adjacent to the coast. Two residential villages, Tok Muda and Sungai Serdang are situated along the same coast and have a greater intake of seafood because of their fishing heritage. The power plant uses seawater as the source for cooling water and the sea is used as a pathway for the transport of coal. At the same time, the surrounding coastal area is the ultimate recipient of the fly ash which is produced by coal burning. As a result, this area has been selected for this study.

### Sampling

Sampling was carried out on August 2008, December 2008 and February 2009. Samples of seafood (fish, shrimp and cockles) popular with the Malaysian population were collected from the fresh catch sold in the local markets of the study area and the catch locations were verified with the fishermen. The seafood types were divided into three groups which are; fish (*Arius maculatus*), crustacea (*Penaeus merguiensis*) and molluscs (*Anadara granosa*). At the same time water samples (25 liters), from six different locations (Figure 1) were collected using a plastic container during each sampling trip. The seafood and water samples were transported to the laboratory for further analysis.

### Analysis of Po^210^

The radiochemical separation method was used to estimate Po^210 ^in the samples [9,10]. The organism samples were dissected to obtain the edible part (muscle) and oven dried at 60°C temperature. About 0.5 g of the dried sample was taken and Po^209 ^of a known activity was added as a yield tracer. Then the samples were digested with nitric acid and perchloric acid. The solution was filtered and gently evaporated to dryness. Then the samples were dissolved in 0.5 M HCl along with a pinch of ascorbic acid to reduce Fe (III) and Po^210 ^was spontaneously deposited on brightly polished silver discs (2 cm diameter) for a period of 3-4 hours at a temperature of 70-90°C. The discs were counted for Po^210 ^activities with an alpha spectrometry system where the detection limit was less than 0.3 Bqkg^-1 ^dry weight. The extraction yield varied from 65 to 80% for the seawater samples and 80 to 95% for the organism samples. Additionally, the combined standard uncertainty of 2σ was calculated involving all the sources of uncertainty. The Po^210 ^deposition was carried out within 2 months of sampling and the activities were calculated at the date of sampling. To ensure the quality of the methodology, Po^210 ^was estimated in a certified reference material IAEA-134 (Cockle flesh) and the measured values were under the 95% confidence interval (mean 4.8 Bqkg^-1^).

About 25 litres of water samples were filtered through pre-weighted Whatman^® ^cellulose filter paper (pore size 0.45 μm). The filtered water was acidified with concentrated nitric acid (HNO_3_) and maintained a pH ≤2. Then about 0.1 ml of 25 mgl^-1 ^Fe^3+ ^as carrier and 0.05 ml of 0.45 Bq ml^-l ^Po^209 ^as yield tracer were added into the water samples. After that, Na_2_CO_3 _was added into the sample and precipitated with ammonium hydroxide (NH_4_OH). The iron (II) hydroxide [Fe(ll)(OH)_2_] precipitate was dissolved with nitric acid and perchloric acid (HClO_4_). After heating the solution for 15 minutes, NH_4_OH was added to maintain the pH 8 and centrifuged in order to obtain a solid Fe(OH)_3 _precipitate. The precipitated residual was dissolved in HClO_4 _and dried at a temperature of 70°C. It was then dissolved in 80 ml 0.5 M HCl, plated and counted according to the above-mentioned method. The analysis of total suspended solids was carried out using the same method as was applied to organism samples.

## Results

**Figure 2:**
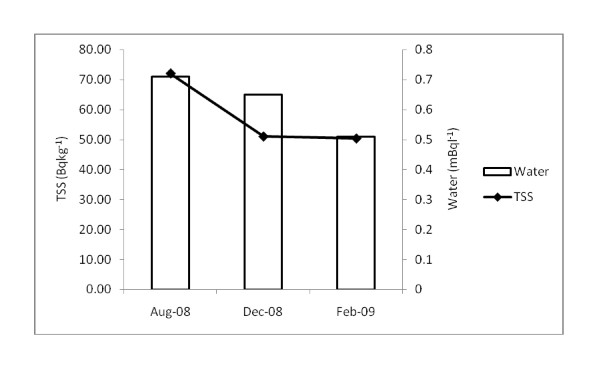
**Concentrations of Po**^**210 **^**in the dissolved and particulate phases of seawater**.

The mean Po^210 ^concentrations measured in dissolved and particulate phases of seawater ranged from 0.51 ± 0.21 to 0.72 ± 0.24 mBql^-1 ^and 50.34 ± 11.40 to 72.07 ± 21.20 Bqkg^-1 ^respectively (Figure 2). Table 1 presents the Po^210 ^concentration found in the seafood samples, collected during three sampling periods. The data shows a significant difference in Po^210 ^concentrations between the three groups of seafood analyzed. The highest values of Po^210 ^activity concentrations were found in molluscs (ranging from 104.3 ± 3.44 to 293.8 ± 10.04 Bqkg^-1^), the lowest values were found in fish (ranged from 4.4 ± 0.12 to 6.4 ± 0.95 Bqkg^-1^) and the middle position was occupied by crustaceans (ranged from 45.7 ± 0.86 to 54.4 ± 1.58 Bqkg^-1^).

**Table 1:** Activity of Po^210 ^in seafood samples collected during three sampling periods

Species	Size class	**Po**^**210 **^**activity (Bqkg**^**-1 **^**dry weight)**		
		
		27/8/2008	2/12/2008	25/2/2009
(Fish) *Arius maculatus*	class 1 (10-15 cm)	5.6 ± 1.78 (n = 5)	NA	3.5 ± 0.14 (n = 9)
	
	class 2 (16-20 cm)	5.9 ± 1.52 (n = 10)	4.5 ± 0.01 (n = 1)	4.5 ± 0.24 (n = 15)
	
	class 3 ( > 20 cm)	7.5 ± 1.64 (n = 15)	5.4 ± 0.27 (n = 29)	5.2 ± 0.24 (n = 6)
	
	Mean	6.4 ± 0.95	5.1 ± 0.13	4.4 ± 0.12

(Crustacean) *Penaeus merguiensis*	class 1 (5-7 cm)	58.2 ± 2.80 (n = 10)	41.5 ± 1.73 (n = 3)	36.8 ± 0.78 (n = 5)
	
	class 2 (8-10 cm)	51.1 ± 2.00 (n = 6)	50.0 ± 3.72 (n = 23)	48.3 ± 1.65 (n = 7)
	
	class 3 (10 > cm)	53.9 ± 3.25 (n = 14)	64.5 ± 1.64 (n = 4)	52.1 ± 1.82 (n = 18)
	
	Mean	54.4 ± 1.58	51.6 ± 1.47	45.7 ± 0.86

(Mollusc) *Anadara granosa*	class 1 (2-3 cm)	112.9 ± 7.24 (n = 3)	224.5 ± 15.0 (n = 16)	114.9 ± 7.29 (n = 15)
	
	class 2 (3.1-3.5 cm)	374.6 ± 13.56 (n = 6)	307.1 ± 20.21 (n = 11)	92.7 ± 2.83 (n = 4)
	
	class 3 (3.5 > cm)	393.9 ± 25.90 (n = 21)	203.0 ± 5.44 (n = 3)	105.2 ± 6.73 (n = 11)
	
	Mean	293.8 ± 10.04	244.9 ± 8.58	104.3 ± 3.44

NA: Not available

n: amount of sample

Po^210 ^uptake from the water column was estimated using the biological concentration factor (BCF) for the marine organisms analyzed in this study [11]. The BCF is the ratio of the Po^210 ^concentration in an organism and the Po^210 ^concentration in the water column. Therefore the internal concentration equals the concentration in water times the BCF value. In general, the concentration factor value is used as transfer parameters in assessments of the public dose of radioactivity in the marine environment [12]. In this study the BCF were calculated on the basis of the values for Po^210 ^activities measured in organism and water samples. In this case the following equation was used, [13](1)

Generally a higher concentration factor (8.5 × 10^4^) was found in the mollusc *Anadara granosa *(Table 2). The concentration factor values for fish and crustaceans were 0.3 × 10^4 ^and 1 × 10^4 ^respectively.

**Table 2:** Calculated data: Concentration Factor, Daily Intake, Committed Effective Dose and Cancer Risk

				Cancer Risk	
				
Species name	CF	**Daily Intake (mBqday**^**-1**^**person**^**-1**^**)**	**CED (μ Svyr**^**-1**^**)**	Lifetime Mortality risk	Lifetime Morbidity risk
*Arius maculatus*	0.3 × 10^4^	199.86	44.69	2.3 × 10^-4^	3.2 × 10^-4^

*Penaeus merguiensis*	1.0 × 10^4^	653.02	186.03	7.7 × 10^-4^	10.6 × 10^-4^

*Anadara granosa*	8.5 × 10^4^	5398.67	517.19	64.3 × 10^-4^	88.2 × 10^-4^

Mean	3.3 × 10^4^	2083.85	249.30	24.8 × 10^-4^	34 × 10^-4^

The daily intake of Po^210 ^is considered to be an accumulation of Po^210 ^in the human body through the consumption of seafood. Taking into account the annual fish landings of 1,055,288 tonnes in 2008 [14] and a total adult population (15 to 65 years old) of 16.88 million in 2005 [15], the daily intake of Po^210 ^via the consumption of seafood was calculated using the following Equation (2); [16](2)

AV is the average concentration (Bqkg^-1 ^fresh weight), AP is the Annual Production, 0.6 is the rate of the edible part, MP is the Malaysian Population and 365 indicate the time. Calculated values of per capita daily intake for fish, crustacean and molluscs are presented in Table 2. A higher value of daily intake was calculated for *Anadara granosa *(5398.67 mBqday^-1^person^-1^) and a lower value for *Arius maculatus *(199.86 mBqday^-1^person^-1^).

The dose received by an adult due to seafood consumption was calculated using the Po^210 ^concentration results measured in analyzed seafood products during this study. Dose calculation for intake of radionuclide by ingestion is based on a dose coefficient taken from the Federal Guidance Report no. 11, EPA. The committed effective dose was calculated as per the following method, [17](3)

D is the annual committed effective dose (μSvyr^-1^); DF is the committed effective dose conversion factor, 1.2 × 10^-6 ^SvBq^-1 ^[18]; MF is the modifying factor (0.6) due to decay of Po^210 ^between catch and consumption [19]; Ai is the Po^210 ^concentration (Bqkg^-1 ^fresh weight); Ci is seafood consumption (kgyr^-1^), per capita seafood consumption for Malaysia was 45.1 kgyr^-1 ^[20] and *f*i is the real fraction consumed (70% for fish, 90% for shrimp and 30% for cockle). The ingestion dose from the consumption of fish, crustacea and molluscs was estimated to be 44.69, 186.03 and 517.19 μSvyr^-1^, respectively (Table 2).

**Figure 3:**
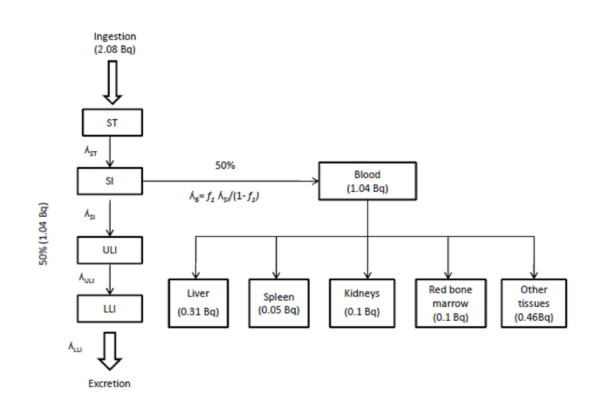
**Activity of ingested Po**^**210 **^**in the internal organs (Based on ICRP 30 and 68)**.

The ICRP recommended in publication 30 [21] the use of the Gastrointestinal tract model to calculate the distribution of radioactive transformation in the body. This model divides the gastrointestinal tract to four compartments such as stomach (ST), small intestine (SI), upper large intestine (ULI) and lower large intestine (LLI). The absorption of ingested material into the blood is generally assumed to occur only in the SI (Figure 3). This absorption is described in terms of a fraction *f*_*1 *_where *f*_*1 *_is the fraction of ingested material that moves from SI to the blood and the fraction 1- *f*_*1 *_moves from SI to ULI. When *f*_*1 *_is very small, most of the activity will pass through the gastrointestinal tract but when *f*_*1 *_is large, most of the activity ingested will be transferred to the systematic circulation. The ICRP report 67 [22] assumed an *f*_*1 *_of 0.5 for Po^210 ^incorporated into food. In this case, it can be assumed that 50% of the ingested Po^210 ^will pass through the GI tract and 50% to the blood. But in the ICRP publication 68 [23] reported that 30%, 10% 5%, 10% and 45% of Po^210 ^leaving the transfer compartment are assigned to liver, kidneys, spleen, red bone marrow and other tissues respectively. In the case of the present study, the internal pathway of Po^210 ^was calculated based on the daily intake value of 2.08 Bq and it was revealed that about 1.04 Bq of Po^210 ^can be transferred through the internal organs. In this way a significant amount of Po^210^can be located in the liver (0.31 Bq), spleen (0.05 Bq), kidney (0.1 Bq), red bone marrow (0.1 Bq) and other internal organs (0.46 Bq). The lifetime cancer risk, R is calculated according to the following equation, [24](4)

r is the cancer risk coefficient and I is the average lifetime intake of Po^210^. Taking into account the average Malaysian life expectancy at birth of 73.39 years [25], the life time intake of Po^210 ^via seafood consumption was calculated from the daily intake. The cancer risk coefficients of Po^210 ^was 4.44 × 10^-8 ^riskBq^-1 ^and 6.09 × 10^-8 ^riskBq^-1 ^for mortality and morbidity risk respectively [26]. The calculated values of mortality risk ranged from 2.3 × 10^-4 ^to 64.3 × 10^-4 ^whereas the morbidity risk varied between 3.2 × 10^-4 ^to 88.2 × 10^-4 ^(Table 2). In both cases the higher risks were associated with mollusc.

## Discussion

The results demonstrated that 99% of the total Po^210 ^activity in the surface seawater of the Kapar coastal area was derived from the particulate phase. A similar trend of Po^210 ^accumulation patterns in water samples was revealed for the North coast of Wales [27] and Kuala Selangor of Malaysia [28]. However, a significant correlation (r = 0.479) was observed between the dissolved and particulate phase. Therefore, it can be assumed that the Po^210 ^in the dissolved and particulate phase were from the same source. The average activity observed in the dissolved phase was 0.62 ± 0.13 mBql^-1 ^which is higher than that of other places in Malaysia [16,28,29]. This higher activity is assumed to be the impact of coal burning in this area. In the suspended particulate matter, Po^210 ^displayed an average concentration value of 57.81 ± 12.35 Bqkg^-1 ^(dw). At various sites in the Western English Channel, the concentration of Po^210 ^in suspended mater varies from 43.1 ± 14.3 to 56.9 ± 15.9 Bqkg^-1 ^[17]. The result of this present study was slightly higher than these reported values. Furthermore, the activity concentration of Po^210 ^in the precipitation and fry ash samples, collected from the same location, ranged from 0.34 to 61.39 mBql^-1 ^and 34.52 to 934.99 Bqkg^-1 ^respectively, which are 2-5 times higher than the normal sampling environment [30]. Therefore it can be assumed that the coastal area of Kapar is affected by the coal burning activity.

Mishra et al. [24] reported that, Po^210 ^was non-uniformly distributed within the Mumbai coastal ecosystem; higher values were associated with molluscs and lower values with crustacea and fish. These results support the present study. The differences in the level of Po^210 ^accumulation in different groups of seafood could be due to the differences in metabolism and feeding pattern. In this study the highest Po^210 ^accumulator species, *Anadara granosa *is a filter feeder and feeds by straining suspended matter and food particles from the water. Additionally, this species has direct contact with sea sediment and this mode of life may contribute to higher levels of Po^210^. On the other hand, the other two species are more mobile and consume food from the water column. Thus these two species demonstrated lower Po^210 ^accumulation. The lowest Po^210 ^accumulator species *Arius maculatus *is a demersal species, distributed within the depth range of 50 to 100 m and feeds on invertebrates and small fishes. On the other hand the middle position is occupied by *Penaeus merguiensis*, which lives in shallow water between 10 and 45 meters on muddy bottoms. This study revealed a clear relationship between Po^210 ^accumulation in organisms and the ecological niche of organisms where the accumulation decreases with depth. Therefore it is assumed that the Po^210 ^accumulation in organisms is regulated by the atmospheric deposition of fly ash. However, shrimps are opportunistic feeders and indiscriminately take food from the benthic zone [31]. Other studies indicate that for the penaeid shrimp, the most important food items are crustacea, molluscs, polychaetes and other benthic invertebrates [32-35], which are also rich in Po^210^. As a result, Po^210 ^concentration in this species is higher than that of fish. Moreover, the edible part of the cockle includes the digestive gland, which is a good accumulator of Po^210^, while in the case of fish and shrimp, only the muscles were consumed. That is why the activity of Po^210 ^is higher in cockles. However, the Po^210 ^concentration in cockles and fish are relatively low in comparison to the values reported in the coastal area of Kuala Selangor, Malaysia [13,16]. The measured values of Po^210 ^concentration were compared with world-wide reported values and presented in Table 3. A comparatively higher level of Po^210 ^concentration was observed in India is because of the impact of a nuclear power station which operates at Kalpakkam. In the case of Cuba, the elevated amount of Po^210 ^activities was characterised by global fallout. Similarly, very high values of Po^210 ^were observed in USA and Australia.

**Table 3:** Po^210 ^concentrations in the muscles of various seafood from different regions of the world.

Species	**Po**^**210 **^**(Bqkg**^**-1 **^**wet weight)**	Locations	References
Sea fish	0.06-5.64	Malaysia	Present study
	
	5-89	Cuba	[9]
	
	0.2-27.48	Syria	[39]
	
	0.6-2.6	Japan	[40]
	
	0.9-5	Poland	[42]
	
	0.4-153.3	USA	[43]
	
	0.25-2.52	Sudan	[50]
	
	0.5-5.3	Brazil	[53]
	
	0.22-4.4	England	[58]
	
	21.4-137.7	India	[59]
	
	0.35-0.90	Baltic-North sea estuary	[60]
	
	0.9-44.10	Australia	[61]

Crustacean	0.65-30.72	Malaysia	Present study
	
	50-151	Cuba	[9]
	
	1.1-35	England	[58]
	
	181.3 ± 7.4	India	[59]
	
	31	Plymouth	[62]

Mollusc	4.61-239.72	Malaysia	Present study
	
	21-30	Cuba	[9]
	
	16-36	England	[58]
	
	305.4- 596.6	India	[59]
	
	29	Ribble estuary	[63]

In general, it is possible to observe the variability of Po^210 ^in seafood depending on different sampling periods and maintaining a good agreement with Po^210 ^activity concentrations in water. Malaysian weather is characterized by two monsoon regimes, the Southwest Monsoon from May to September, and the Northeast Monsoon from November to March. Po^210 ^activity varies slightly between the monsoons in this study. In general, Po^210 ^activity in the study area was higher in August 2008 (southwest monsoon). During this time the wind flow is usually light (below 15 knots) [36]. Thus the fly ash from the power plant gets enough time to mix into the surrounding area, resulting in a higher Po^210 ^activity. On the other hand, during the northeast monsoon, when there is a strong wind flow of usually more than 30 knots [36], the fly ash from the power plant is quickly dispersed and therefore the activity in the surrounding area is low.

Po^210 ^concentration in mussels is reportedly size-dependent [37]. Surprisingly, in this study it is very clear that Po^210 ^accumulation in seafood is not dependent on the organisms' size. As a result it can be deduced that Po^210 ^in the organisms included in this study is mostly associated with environmental conditions. An analysis using Pearson's correlation coefficient indicates a statistically significant linear relationship between the Po^210 ^concentration and the total weight of cockles (r = 0.62, p < 0.01), whereas the correlation between Po^210 ^and total length was low (r = 0.37, p < 0.01). There was a week correlation between Po^210 ^and total length in fish (r = 0.29, p < 0.01). However, we failed to discover any correlations for shrimp. The variation is because of the ecological niche of these species where cockles are stagnant in one place but the other two species are mobile. Thus, due to differences in grazing habits, the influence of differing environmental concentrations on fish and shrimp is comparatively substantial compared to that of cockles.

**Figure 4:**
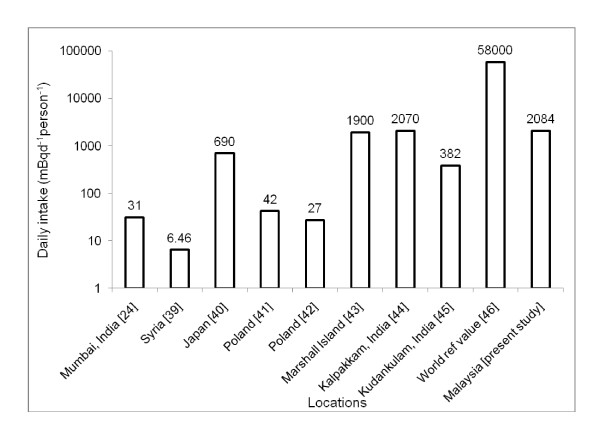
**A comparison of daily intake of Po**^**210 **^**in different locations around the world**.

The highest value of concentration factors was associated with molluscs and the lowest with fish. In previous studies, lower concentration factors in fish compared to molluscs' tissues were observed at Kuala Selangor, Malaysia [13,16]. Therefore, it can be concluded that molluscs can be considered as a higher contributor of Po^210 ^exposure to the seafood-consuming populace in the study area. The observed concentration factor values obtained for molluscs and fish were comparatively higher than the values published by the IAEA, [38] which are 2 × 10^3 ^for fish, 2 × 10^4 ^for crustaceans and molluscs. Mean daily intake values were compared with reported values in other countries (Figure 4) and values observed in this study were found to be relatively higher [24,39-45]. On the other hand, the daily intake for the Malaysian population was lower than that of the world reference value [46]. However, the elevated value of daily intake for Malaysia may be due to the reliance on seafood as the main protein source among Malaysians and higher Po^210 ^concentrations in the edible portion of seafood, which could be due to the impact of coal burning.

**Figure 5:**
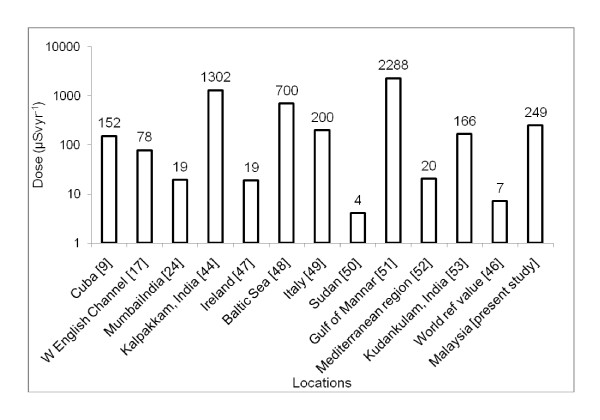
**A comparison of CED in different locations around the world**.

Due to higher Po^210 ^activity concentrations in molluscs and crustaceans, the committed effective doses for these two species were comparatively higher than in fish. The average committed effective dose can be compared to those published elsewhere regarding the ingestion of Po^210 ^via seafood consumption (Figure 5). The value of the present study is within the range of other places [9,17,24,44,47-53] but much higher than the world reference value [46]. On the other hand, in India, foods of animal origin, especially crabs, fish and prawn deliver significantly greater doses (93 to 3364 μSvyr^-1^) to the public [44]. However, the average dose (0.25 mSvyr^-1^) is lower than the limit (1 mSvyr^-1^) described in the ICRP-2007 recommendation [54]. It is reported that the global average of annual radiation doses from natural radiation sources is 2400 μSv [55]. Therefore, according to the data in the present study, the consumption of seafood can single-handedly contribute up to 10.38% of natural radioactivity to the public which is not a negligible amount.

On the other hand, Nielsen et al.[48] studied artificial radionuclide (Cs-137 and Sr-90) in the Baltic Sea marine environment and for a critical group, the maximum dose in humans was estimated to be 40-200 μSvyr^-1^. If these values are compared with the present study, it can be observed that, in spite of it being a naturally occurring radionuclide, Po^210 ^alone is contributing to a higher exposure than that of artificial radionuclide. Therefore, it is very clear that this unusually higher level of radiation is the result of the coal burning and the higher level of Malaysian seafood consumption. In the case of the present study, we tried to apply the concept to the ICRP model and discovered that a significant amount of Po^210 ^can be allocated to the internal organs of seafood consumer.

Among the three species analyzed in this study, molluscs play a significant role as a higher risk contributor to the public. The average mortality and morbidity risk was found to be 24.8 × 10^-4 ^and 34 × 10^-4 ^correspondingly. Usually, the US-EPA considers cancer risks that are bellow 1 × 10^-6^, to be so small as to be negligible and risks above 1 × 10^-4 ^to be adequately large that some sort of remediation is desirable. Cancer risks that range between 1 × 10^-6 ^and 1 × 10^-4 ^are generally considered to be acceptable [56,57]. Therefore, from the calculated values obtained in this study, the seafood-consuming populace may be at considerable risk.

## Conclusions

This study provides a general view of α-emitting radionuclide Po^210 ^in the Kapar power plant area. The concentration of Po^210 ^in seawater samples was comparatively higher than other places. Po^210 ^was non-uniformly distributed with the groups of organisms. The accumulation of Po^210 ^in the seafood was not related to the body size but was found to be strongly variable with ambient water concentrations and seasonal changes. The calculated values of concentration factors for molluscs and fish were higher than the standard values dictated by the IAEA. The daily intake and dose of Po^210 ^due to the consumption of seafood was also calculated and found to be within the range of other studies. Based on the concept of ICRP, a preliminary conservative method was designed to calculate the internal organ dose and it was found that a very significant amount of Po^210 ^can be allocated to the internal organs of the Malaysian seafood consumer. Besides that, the calculated cancer risks of this study also crossed the limits recommended by US-EPA. Results presented here suggest that the area around the Kapar Power station is susceptible to some contamination and people living near the area are exposed to higher alpha radiation through seafood consumption. These findings suggest that appropriate action needs to be done to mitigate possible risks to human and environmental health.

## Abbreviations

IAEA: International Atomic Energy Agency; EPA: Environmental Protection Agency; ICRP: International Commission on Radiological Protection; TSS: Total Suspended Solids.

## Competing interests

The authors declare that they have no competing interests.

## Authors' contributions

The study was designed by Mohamed and Lubna. The concentration of Po^210 ^analysis was undertaken by Lubna. The first draft of the manuscript was prepared by Lubna and comments and changes were made by Mohamed. Both authors read and approved the final manuscript.
